# Dielectrophoresis Multipath Focusing of Microparticles through Perforated Electrodes in Microfluidic Channels

**DOI:** 10.3390/bios9030099

**Published:** 2019-08-07

**Authors:** Anas Alazzam, Mohammad Al-Khaleel, Mohamed Kamel Riahi, Bobby Mathew, Amjad Gawanmeh, Vahé Nerguizian

**Affiliations:** 1 Mechanical Engineering Department, Khalifa University, Abu Dhabi 127788, UAE; 2 Electrical Engineering Department, École de Technologie Supérieure, Montreal, Quebec, QC H3C 1K3, Canada; 3 Department of Applied Mathematics and Sciences, Khalifa University, Abu Dhabi 127788, UAE; 4 Department of Mathematics, Yarmouk University, Irbid 21163, Jordan; 5 Mechanical Engineering Department, United Arab Emirates University, Al Ain 15551, UAE; 6 Electrical and Computer Engineering Department, Khalifa University, Abu Dhabi 127788, UAE; 7 Electrical and Computer Engineering Department, Concordia University, Montreal, Quebec, QC H3C 1K3, Canada

**Keywords:** dielectrophoresis, perforated electrodes, sorting, focusing, microfluidics, microparticles, microchannel, DEP, LoC, Bio MEMS

## Abstract

This paper presents focusing of microparticles in multiple paths within the direction of the flow using dielectrophoresis. The focusing of microparticles is realized through partially perforated electrodes within the microchannel. A continuous electrode on the top surface of the microchannel is considered, while the bottom side is made of a circular meshed perforated electrode. For the mathematical model of this microfluidic channel, inertia, buoyancy, drag and dielectrophoretic forces are brought up in the motion equation of the microparticles. The dielectrophoretic force is accounted for through a finite element discretization taking into account the perforated 3D geometry within the microchannel. An ordinary differential equation is solved to track the trajectories of the microparticles. For the case of continuous electrodes using the same mathematical model, the numerical simulation shows a very good agreement with the experiments, and this confirms the validation of focusing of microparticles within the proposed perforated electrode microchannel. Microparticles of silicon dioxide and polystyrene are used for this analysis. Their initial positions and radius, the Reynolds number, and the radius of the pore in perforated electrodes mainly conduct microparticles trajectories. Moreover, the radius of the pore of perforated electrode is the dominant factor in the steady state levitation height.

## 1. Introduction

In the last decade, several researchers and companies started looking at miniaturized technologies (Bio MEMS) for biological particle manipulation, analysis, detection and characterization [1,2,3,4,5,6,7,8]. Researchers from different fields of engineering have developed different microsystems for a wide variety of applications [9,10] and this includes separation, switching, and focusing of cells and particles [11,12,13,14,15]. The recent achievements in creating different types of microfluidic devices have been made possible by performing complex biological operations in a short time and using very small amounts of reagents. The ultimate objective of research was to reproduce the same laboratory biological assays with miniaturized Bio MEMS devices. Researchers started to create miniaturized devices called Lab-on-chip (LoC) capable of performing several operations of detection and analysis on exosomes, micro vesicles, and circulating tumor cells. These LoC devices achieved active sorting and separation using dielectrophoresis (DEP) based on the dielectric properties of particles or vesicles [16,17,18,19,20]. The transfer from LoC to Point-of-Care (PoC) diagnostics requires multi parameter testing. This will require several steps, from separation, focusing, detection, and many other transport processes for proactive diagnosis/prognosis and therefore, DEP has a crucial role in the abovementioned processes. An important key sub-operation in creating microfluidic systems was the centering or focusing of microparticles enabling the isolation and sequencing of each individual particle for detection and screening purposes. Focusing, in general, is a pre-step for switching and separation and is defined as sorting of randomly distributed cells and particles [21]. Focusing microparticles by restricting their motion to a horizontal or vertical plane is defined as 2D focusing. Such focusing is achieved by subjecting the microparticles to coplanar forces. Alternatively, focusing the microparticles by confining their motion into a single line is defined as 3D focusing. The microparticles could be 3D focused if the summation of all the applied forces is zero at a single point in one plane and makes a straight line in the plane perpendicular to that plane. Several focusing techniques are reported in the literature [21,22,23,24,25,26]. Dielectrophoresis has been reported for 2D and 3D focusing of microparticles [27,28]. DEP is defined as the motion of particles suspended in conductive medium under the effect of forces created by non-uniform electric field [29,30]. The microparticles’ movement will be toward the high electric field gradient if the particles are more polarizable than the surrounding medium. This phenomenon is called positive DEP (or pDEP). On the other hand, if the microparticles are less polarizable than the medium, it will move toward the low electric field gradient in a phenomenon called negative DEP (nDEP) [31,32,33]. The mathematical expression for the DEP force acting on a microparticle is given by Equation (1) [29,34].

(1) $$F_{DEP}=2\pi \varepsilon_{m}r_{e}^{3}Re\left[f_{CM}\right]\nabla E_{RMS}^{2}$$

(2) $$f_{CM}=\frac{\left(\varepsilon_{e}-\frac{\sigma_{e}}{\omega }j\right)-\left(\varepsilon_{m}-\frac{\sigma_{m}}{\omega }j\right)}{\left(\varepsilon_{e}-\frac{\sigma_{e}}{\omega }j\right)+2\left(\varepsilon_{m}-\frac{\sigma_{m}}{\omega }j\right)}$$

where the constants *ε_m_* (F/m), *r_e_* (m), *f_CM_* are the permittivity of the medium, radius of the microparticle, and the Clausius–Mossotti factor, respectively. The electric field experienced by the microparticle is *E_RMS_* (V/m) and the angular frequency of the signal is ω (rad/s). The values *σ_m_* (S/m), *σ_e_* (S/m), and *ε_e_* (F/m) are the conductivity of the medium, and the conductivity and permittivity of the microparticle, respectively. The Clausius–Mossotti factor in Equation (2) is function of ω for particular microparticles and medium. The response of particles to the electric field (nDEP to pDEP) could be changed by altering the frequency of the signal ω. 

**Figure 1:**
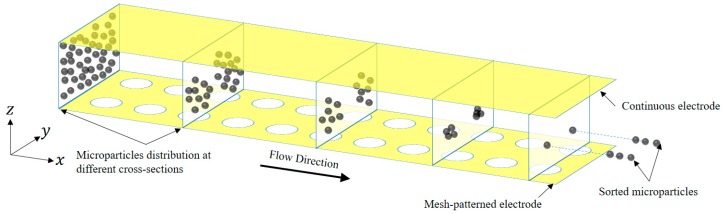
Schematic of the microfluidic device showing the distribution of microparticles at different cross-sections along the channel. The microchannel has one continuous electrode on top surface and a mesh-patterned electrode on the bottom part of the channel. Yellow color represents the electrodes.

The non-uniform electric field created by the electrodes directly influences the DEP force as demonstrated by Equation (1). On the other hand, each electrode structure is responsible for creating an electric field in the dedicated channel. As a result, the collection of electrodes in a particular configuration or pattern results in a specific electric field, and changing it produces a different electric field. This tool has been used in the literature for controlling the movement of microparticles in the channel [5,23]. This research introduces a novel electrode configuration that is designed to achieve 3D focusing of microparticles with different stream of paths as illustrated in Figure 1. The design is based on two layers of electrodes separated by the height of the channel, a top continuous layer, and a bottom one designed with circular mesh patterns. This configuration resulted in 3D focusing throughout the microchannel at various paths, as shown in Figure 1. Such a system can be implemented in the lab without any complications as opposed to other 3D electrodes that have been previously proposed [35,36].

In this work, a mathematical model is first presented capturing the electric field in a microchannel under the effect of electrodes in a specific configuration. This model is then used to show how 3D focusing can be achieved with such a configuration. To the best of our knowledge, there is no single mathematical model that can capture all DEP forces in a microchannel under various electrodes configurations.

Lin et al. [37] developed a microfluidic device that employs both sheath flows and DEP for achieving 3D focusing of microparticles. Sheath flow is initially used for focusing the microparticles in a vertical plane passing through the middle of the microchannel. These microparticles in the vertical plane are then subjected to a nDEP force in the vertical direction, using planar electrodes located on the top and bottom surfaces of the microchannel, to 3D focus the microparticles at the center of the microchannel. Holmes et al. [5] created a microfluidic device for DEP-based 3D focusing of microparticles. The device has four planar trapezoidal electrodes with two electrodes on the top and bottom surfaces of the microchannel. The electric field in this microfluidic device is created between each of the top and bottom electrodes, and this helps realize nDEP forces that focus microparticles at the center of the microchannel. Yu et al. [36] also developed a microfluidic device for 3D focusing of microparticles using DEP. This device has multiple finite-sized circular interdigitated transducer (IDT) electrodes in the inner surface of the microfluidic device. Each electrode and its neighboring electrodes generate electric field thereby creating nDEP force that focuses the microparticles at the center of the microfluidic device. Wang et al. [35] fabricated a microfluidic device that has vertical IDT electrodes on both the sidewalls. The electrode configuration subjects microparticles to the nDEP forces from both the sides which allows focusing them at the center of the microchannel.

In this paper, a theoretical model is developed for a circular mesh-based electrode configuration that can achieve focusing of microparticles in 3D with different paths. The proposed system can perform focusing at multiple target locations at the same time, where the path followed by the particle is decided based on its initial conditions and the electric field of the electrodes. This system can be easily implemented in the lab since the electrodes are located orthogonal to the sidewalls.

## 2. Approach and Method

The mathematical model (Supplementary Materials) of the proposed microfluidic device is presented based on assumptions that are listed in Mathew et al. [26]. The model consists of the equation of motion, continuity equation, Navier–Stokes equation, Laplace equation representing electric potential and electric field. The equation of motion is provided below [38].

(3) $$m_{e}\frac{d^{2}}{dt^{2}}\left[\begin{array}{c} x_{e}\\ y_{e}\\ z_{e} \end{array}\right]=\left[\begin{array}{c} \sum^{\;}F_{ext,x}\\ \sum^{\;}F_{ext,y}\\ \sum^{\;}F_{ext,z} \end{array}\right]$$

where *m_e_* (kg) is the mass of the microparticle, *x_e_* (m) is the displacement of the microparticle in the *x*-direction, *y_e_* (m) is the displacement of the microparticle in the *y*-direction, *z_e_* (m) is the displacement of the microparticle in the *z*-direction, and *F_ext_* is the sum of all external forces acting on the microparticle. The term on the left-hand side of Equation (3) represents the inertial force. The external forces acting on the microparticle include that due to buoyancy, drag, and dielectrophoresis. The forces are represented by Equations (4) and (5), respectively [39].

(4) $$\left[\begin{array}{c} F_{g,x}\\ F_{g,y}\\ F_{g,z} \end{array}\right]=V_{e}\rho_{e}\left[\begin{array}{c} 0\\ 0\\ -g \end{array}\right]$$

(5) $$\left[\begin{array}{c} F_{b,x}\\ F_{b,y}\\ F_{b,z} \end{array}\right]=V_{e}\rho_{m}\left[\begin{array}{c} 0\\ 0\\ g \end{array}\right]$$

The force associated with drag is provided in Equation (6) [39].

(6) $$\left[\begin{array}{c} F_{d,x}\\ F_{d,y}\\ F_{d,z} \end{array}\right]=6\pi \mu_{m}r_{e}\left[\begin{array}{c} u_{m,x}-u_{e,x}\\ u_{m,y}-u_{e,y}\\ u_{m,z}-u_{e,z} \end{array}\right]$$

where *F_d_* (N) is the drag force, *μ_m_* (Pa.s) is the viscosity of the medium, *u_m_* (m/s) is the velocity of the medium, and *u_e_* (m/s) is the velocity of the microparticle. For microfluidic devices handling microparticles, the volumetric flow rate is low such that the flow in these devices is fully developed. Under fully developed flow conditions, the medium velocity in the transverse directions is zero. The velocity of the medium in rectangular microchannels along the axial direction is represented by Equation (7) [29].

(7) $$u_{m,x}=\frac{48{\dot{Q}}_{m}\sum_{i=1,3,5}^{\infty }\left(\frac{{\left(-1\right)}^{\left(\frac{i-1}{2}\right)}}{i^{3}}\right)\cos\left[\frac{i\pi }{W_{ch}}\left(y-\frac{W_{ch}}{2}\right)\right]\left\{1-\frac{\cosh\left[\frac{i\pi }{W_{ch}}\left(z-\frac{H_{ch}}{2}\right)\right]}{\cosh\left(\frac{i\pi }{2}\frac{H_{ch}}{W_{ch}}\right)}\right\}}{\pi^{3}W_{ch}H_{ch}\left[1-\frac{192\;W_{ch}}{\pi^{5}H_{ch}}\overset{\infty }{\underset{i=1,3,5}{\sum }}\frac{\tanh\left(\frac{i\pi }{2}\frac{H_{ch}}{W_{ch}}\right)}{i^{5}}\right]}$$

where *W_ch_* (m) and *H_ch_* (m) are the width and the height of the microchannel, respectively, ${\dot{Q}}_{m}$ (m^3^/s) is the volumetric flowrate, *z* is the coordinate in the vertical direction of any location from the bottom corner on the left side of the cross-section and *y* is the coordinate in the horizontal direction of any location from the bottom corner on the left side of the cross-section. To solve Equation (3), two initial conditions are required and these are provided in Equations (8) and (9). The first initial condition refers to the position of the microparticle at the inlet of the microchannel, which is taken to be same as the start of the focusing section in the model, while the second initial condition refers to the velocity of the microparticle at the start to the focusing section.

(8) $$\left[\begin{array}{c} x_{e}^{0}\\ y_{e}^{0}\\ z_{e}^{0} \end{array}\right]=\left[\begin{array}{c} 0\\ y_{o}\\ z_{o} \end{array}\right]$$

(9) $$\left[\begin{array}{c} u_{e,x}^{0}\\ u_{e,y}^{0}\\ u_{e,z}^{0} \end{array}\right]=\left[\begin{array}{c} u_{m,x}\left(y_{o},z_{o}\right)\\ 0\\ 0 \end{array}\right]$$

where $x_{e}^{0}$ (m), $y_{e}^{0}$ (m), and $z_{e}^{0}$ (m) are the initial positions of the microparticle in the *x*, *y*, and *z*-directions, respectively. The initial position $y_{e}^{0}$ is set equal to *y_o_* and the initial position $z_{e}^{0}$ is set equal to *z_o_*. The initial velocities in the three directions (*x*, *y*, and *z*) are $u_{e,x}^{0}$ (m/s), $u_{e,y}^{0}$ (m/s), and $u_{e,z}^{0}$ (m/s). The initial velocity in the x-direction is set equal to the medium’s velocity at *y_e_* = *y_o_* and *z_e_* = *z_o_*.

Equation (3) is solved using finite difference method as detailed in Mathew et al. [26]. The first and second order central difference schemes is used to replace the differential terms in Equation (3). Implementation of the difference schemes to Equation (3) yields Equation (10). The processing time step is taken to be 10 µs.

(10) $$\left[\begin{array}{c} x_{e}^{n+1}\\ y_{e}^{n+1}\\ z_{e}^{n+1} \end{array}\right]=\left[\begin{array}{c} {}[\left(\frac{3\pi \mu_{m}r_{e}\Delta t}{m_{e}}-1\right)x_{e}^{n-1}+2x_{e}^{n}+\\ \frac{3\pi \mu_{m}r_{e}\Delta t^{2}U_{m,x}}{m_{e}}\\ +\frac{2\pi \varepsilon_{m}r_{e}^{3}Re\left[f_{CM}\right]\Delta t^{2}}{m_{e}}\nabla_{x}E_{RMS}^{2})]\\ {}[\left(\frac{3\pi \mu_{m}r_{e}\Delta t}{m_{e}}-1\right)y_{e}^{n-1}+2y_{e}^{n}+\\ +\frac{2\pi \varepsilon_{m}r_{e}^{3}Re\left[f_{CM}\right]\Delta t^{2}}{m_{e}}\nabla_{y}E_{RMS}^{2})]\\ {}[\left(\frac{3\pi \mu_{m}r_{e}\Delta t}{m_{e}}-1\right)z_{e}^{n-1}+2z_{e}^{n}+\\ g\Delta t^{2}\left(\frac{\rho_{m}}{\rho_{e}}-1\right)+\\ \frac{2\pi \varepsilon_{m}r_{e}^{3}Re\left[f_{CM}\right]\Delta t^{2}}{m_{e}}\nabla_{z}E_{RMS}^{2})] \end{array}\right]$$

Equation (10) can be employed for determining displacements when n > 0. When *n* = 0, there is need for knowing the displacement at $x_{e}^{-1}$, $y_{e}^{-1}$, and $z_{e}^{-1}$. These are determined using information on the initial conditions provided in Equations (8) and (9). The electric field is dependent on the electric potential and thus it needs to be determined prior to solving Equation (3). The electric potential inside the microchannel is dictated by Equation (11). The relationship between electric potential and electric field is provided in Equation (12).

(11) $$\Delta \varphi_{RMS}=0$$

(12) $$\left[\begin{array}{c} E_{x,\;RMS}\\ E_{y,RMS}\\ E_{z,RMS} \end{array}\right]=-\left[\begin{array}{c} \frac{\partial }{\partial x}\\ \frac{\partial }{\partial y}\\ \frac{\partial }{\partial z} \end{array}\right]\varphi_{RMS}$$

**Figure 2:**
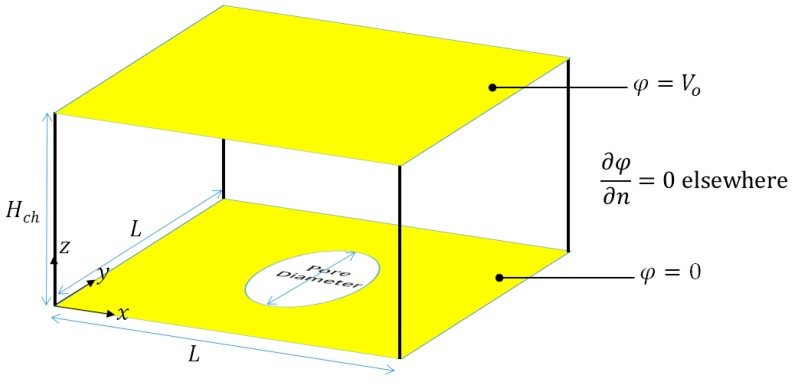
Schematic diagram of the repeating unit showing the boundary conditions and dimensions. The electrodes are shown in yellow.

The solution of Equation (11) is not determined for the entire microchannel, but only over a small portion of the microchannel, which is equivalent to the repeating unit of the microchannel, Figure 2. The boundary conditions associated with Equation (11) are provided in Equation (13). Equation (13.a) represents the voltage applied on one of the electrodes while Equation (13.b) represents the voltage applied on the second electrode.

(13.a) $$\varphi =V_{o}{\;\;\;\;\;\;\;\;\Pi }_{v}\subset \partial \Omega$$

(13.b) $$\varphi =0{\;\;\;\;\;\;\;\;\Pi }_{D}\subset \partial \Omega$$

(13.c) $$\frac{\partial \varphi }{\partial n}=0{\;\;\;\;\;\;\;\;\Pi }_{N}\subset \partial \Omega$$

Finite element method is used for solving Equation (11). The weak formulation of Equation (11) is provided in Equation (14).

(14) $$\int_{\Omega }\nabla \varphi .\nabla \phi dx-\int_{\partial \Omega }\frac{\partial \varphi }{\partial n}.\;\phi d\Pi =0$$

where ϕ is any test function, which is infinitely differentiable with compact support in the volume Ω. Typically, belonging to the Sobolov space $H_{0}^{1}\left(\Omega \right)$. Numerically, the space $H_{0}^{1}\left(\Omega \right)$ will be approximated by the finite dimension space $V_{h}\left(\Omega \right)$, known as the finite element space, which is spanned by {*ϕ_i_*}, *i* = 1, …, *N*. These basis functions will help approximating any function as shown in Equation (15).

(15) $$\varphi_{h}=\;\sum_{i=1}^{N}\alpha_{i}\phi_{i}$$

Note the use of $\varphi_{h}$ instead of φ is to highlight the discrete solution $\varphi_{h}$ that approximates the continuous one φ. Now applying Equations (14) and (15) and using the homogenous Neumann boundary condition in Equation (13) results in Equation (16).

(16) $$\sum_{i,j=1}^{N}\int_{\Omega }\alpha_{i}\nabla \phi_{i}\nabla \phi_{j}dx=0$$

Equation (16) is a linear system with unknown $\alpha =\alpha_{i}\epsilon R^{N}$, of the form shown below.

$$A\alpha =0\;\;\;\;\;\;\;\;A\in R^{N\times N}$$

where $A_{i,j}=\int_{\Omega }\nabla \phi_{i}\nabla \phi_{j}dx$, which is called Stiffness matrix. In practice, we triangulate the volume Ω and consider the test functions $\left\{\phi_{i}\right\}$ as Lagrange polynomials of order 2. The Dirichlet boundary condition is taken by enforcement on the linear homogenous system Equation (16) where if $\alpha_{j}$ is the unknown on the homogenous Dirichlet boundary condition, the row j of the matrix A is taken to be zero except its diagonal entry $A_{j,j}$, which is set to be equal to one.

## 3. Results and Discussion

This section demonstrates the numerical analysis for the proposed device to reach ‘3D focusing’ of microparticles simultaneously at multiple locations along the width of the microfluidic device as well as analyzes the effect of different geometric and operating parameters on 3D focusing. For demonstration purposes, polystyrene is used as the microparticle and the medium employed is water. All associated properties are available in Mathew et al. [40]. Additionally, microparticles exhibit nDEP when the frequency of the AC signal is very high, typically above 0.1 MHz. At this operating frequency band, the effect of conductivities on Re[*f_CM_*] can be neglected. It needs to be stressed here that when subjecting cells to nDEP, the operating frequency needs to be small in which case the influence of permittivities on Re[*f_CM_*] cannot be neglected. For all results presented in this section, the operating frequency is maintained greater than 1 MHz.

The steady state location of the particle in the XY plane along the Y-direction depends on the DEP forces experienced from the left and right sides, mathematically this can be expressed by

(17) $${\left(2\pi \varepsilon_{m}r_{e}^{3}Re\left[f_{CM}\right]\nabla E_{RMS}^{2}\right)}_{left}={\left(2\pi \varepsilon_{m}r_{e}^{3}Re\left[f_{CM}\right]\nabla E_{RMS}^{2}\right)}_{right}$$

Equation (17) can be simplified to the form

(18) $${\left(\nabla E_{RMS}^{2}\right)}_{left}={\left(\nabla E_{RMS}^{2}\right)}_{right}$$

As stated in Equation (18), the steady state location of the particles along the Y-direction depends only on the gradient of electric field generated by the electrodes. All other parameters have no effect on the steady state location along Y-axis. Particles of different types and sizes will be sorted to flow above the center of the circular pores. However, the steady state location of the particle along the X-direction depends on two main forces, the DEP force from the electrodes and the drag force from the flow. The forces equation on the particle along the X-axis is:

(19) $$6\pi \mu_{m}r_{e}\left(u_{m,x}-u_{e,x}\right)=2\pi \varepsilon_{m}r_{e}^{3}Re\left[f_{CM}\right]{\left(\nabla E_{RMS}^{2}\right)}_{x}$$

**Figure 3:**
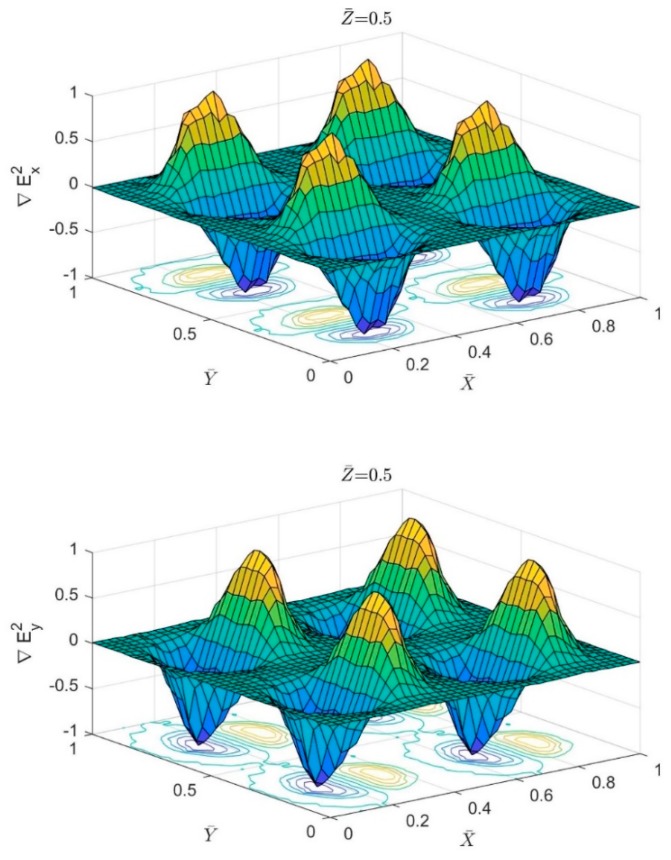
The normalized variation of the term $\nabla E_{RMS}^{2}$ in the XY plane at $\bar{Z\;}$ = 0.5. The first subfigure shows the variation in the X-component while the second one is for the variation in Y-component.

Figure 3 shows the normalized variation in x and y components of$\;\nabla E_{RMS}^{2}$ above four circular pores. It is clear from the figure that particles will reach the steady state location along the Y-axis when they flow above the center of the circular pores where the DEP force from both sides approaches zero. Though the particle will experience fluctuation in the total forces along the X-axis as the DEP force will vary between positive and negative values while the particle passing the circular pores.

The two dominant forces along the Z-axis are the DEP force from the partially perforated electrodes and the sedimentation force. The particles will reach steady state location along Z-direction when both forces are equal. This can be mathematically expressed by the following equation.

(20) $$2\pi \varepsilon_{m}r_{e}^{3}Re\left[f_{CM}\right]\nabla E_{RMS}^{2}=\left(\rho_{e}-\rho_{m}\right)V_{e}g$$

Equation (20) can be simplified to the form

(21) $$3\varepsilon_{m}Re\left[f_{CM}\right]\nabla E_{RMS}^{2}=2\left(\rho_{e}-\rho_{m}\right)g$$

as shown from the above equation, the steady state location of the particle along the Z-direction is independent of the radius of the same. Particles of identical type and different sizes will have the same steady state location along the Z-axis. Particles of different types could be sorted to flow at different levels along the Z-axis.

**Figure 4:**
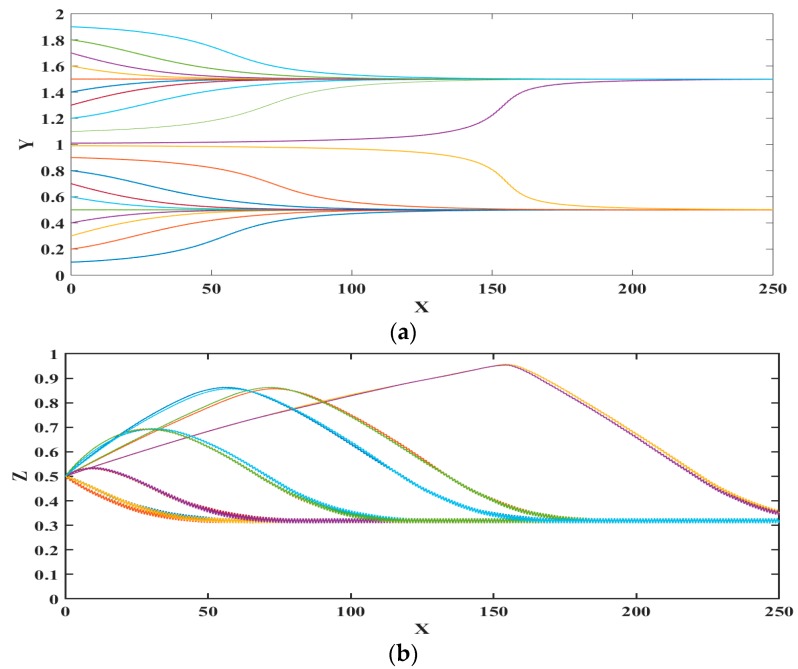
Trajectories of microparticle in the microfluidic device with different initial location along the y-axis (**a**) top view; and (**b**) side view. X, Y, and Z are the dimensionless lengths x/L, y/L, and z/Hch, respectively.

Figure 4 represents the trajectory (non-dimensional displacements) of microparticle for different initial locations along the width of the microchannels; the vertical location is maintained the same. From Figure 4 it can be noticed that polystyrene microparticles are focused at two locations, inside the microchannel, with each being the center of the circular pore. This is because the mesh-patterned bottom electrode creates nDEP force in the horizontal and vertical directions towards the center of each circular pore. As there are two lines of circular pores on the bottom surface, there are two positions or paths to which each microparticle can translate. The preferred location, i.e., circular pore center, of choice for each microparticle depends on its proximity to a circles’ center. The preferred circles’ center for a microparticle is the one that is closest to it. It is stressed here that if the number of circular pores lines is increased, then the number of stable positions to which the microparticles can translate will also proportionally increase.

**Figure 5:**
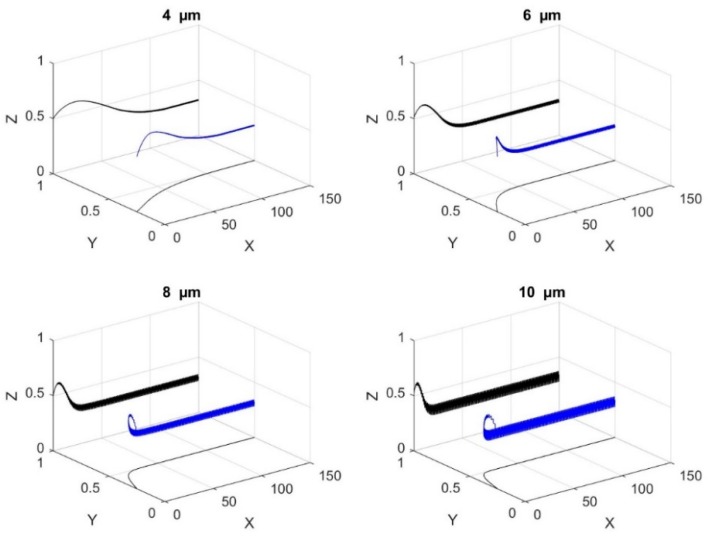
The impact of the size of the microparticle on its trajectory along the microchannel (4 μm, 6 μm, 8 μm, 10 μm). The projections on the planes xy and xz are shown. X, Y, and Z are the dimensionless lengths x/L, y/L, and z/Hch, respectively.

Figure 5 shows the effect of a microparticle radius on 3D-focusing; non-dimensional displacements are provided in this figure. It is clear that the microparticle radius has no effect on the steady state position of the microparticles as stated in Equations (18) and (20); however, the path of the microparticle is influenced by its radius. Both nDEP and sedimentation forces balance each other at steady state position and since both of these forces are function of *r*^3^, the radius of microparticle does not affect the steady state position as stated in Equation (21). From Figure 5 it can be noticed that the axial distance required for the microparticle to reach its steady state location is dependent on its radius; the axial distance reduces with increase in radius. Under transient conditions, the forces are unbalanced and thus dependent on the radius. Figure 5 also shows that microparticle oscillates in the vertical direction, which is due to the variation of the vertical component of the electric field in the axial direction.

The final levitation height is dependent only on the properties of microparticle and the medium as stated in Equation (21). As seen from Figure 5, after reaching steady state, microparticles experience oscillation. The degree of oscillation increases with increase in microparticle radius. Microparticles experience oscillations due to the variation of the DEP force as they flow along the channel as shown in Figure 3. Since the force due to DEP is dependent on microparticle radius, the degree of oscillations increases with increase in the same.

**Figure 6:**
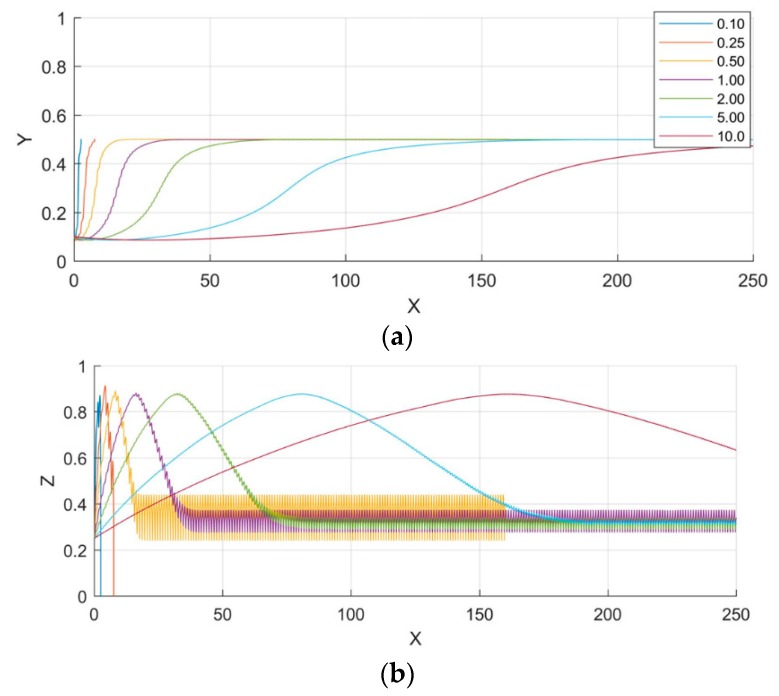
The impact of Reynolds number on the trajectory of microparticles (**a**) top view; and (**b**) side view. The Reynolds numbers are given in the legend of (**a**). The projections on the planes xy and xz are shown. X, Y, and Z are the dimensionless lengths x/L, y/L, and z/Hch, respectively.

Figure 6 shows the influence of Reynolds number (*Re*) of the trajectory of microparticle. It can be noticed that the microparticle cannot be focused for low *Re*. At low *Re*, the drag force acting on the microparticle to move it in the horizontal (axial) direction of the microchannel is smaller than the horizontal nDEP force opposing it due to which the microparticle falls to bottom of the microchannel. Thus, there exists a threshold value of *Re* below which it is not possible to 3D focus microparticles. It can be noticed from Figure 6 that the axial distance travelled by the microparticle before reaching its steady state position increases as *Re* increases. This is because with increase in *Re*, the associated fluid velocity increases which leads to the microparticle traveling greater distance before reaching the steady state location. Also, it can be noticed from Figure 6 that steady state position of microparticles is independent of Re when it is greater than its threshold value. This is obvious since none of the forces acting on the microparticle is influenced by the volumetric flow rate at steady state along Y and Z axes.

**Figure 7:**
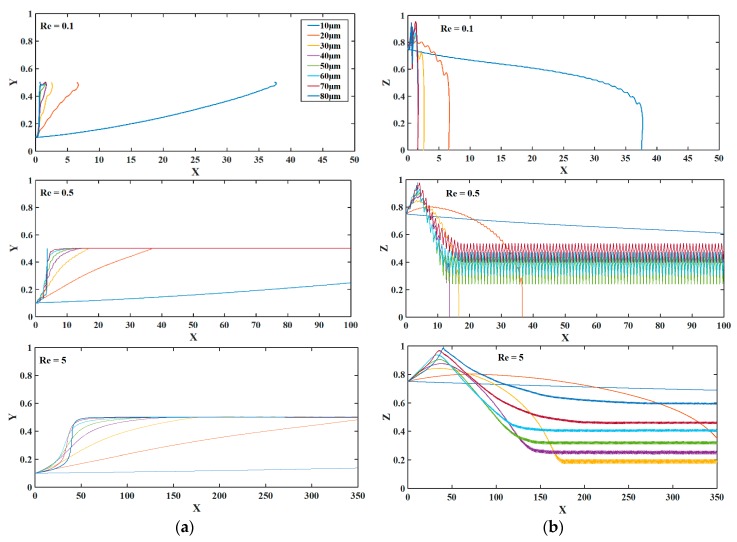
The impact of the pore radius of the perforated surface electrode on the trajectory of microparticle at different Reynolds numbers (**a**) top view; and (**b**) side view. The radius of perforated surface electrode is given in the legend. X, Y, and Z are the dimensionless lengths x/L, y/L, and z/Hch, respectively.

The influence of the radius of the perforated surface is studied and the results are shown in Figure 7. The figure represents non-dimensional displacements. It can be noticed from Figure 7 that at *Re* = 0.1 the microparticle cannot be focused and drop to the bottom of the microchannel. This happens because the drag force experienced by the microparticle is smaller than the horizontal component of the nDEP force opposing it. On the other hand, for higher *Re* the steady-state levitation height increases with increase in the radius of the mesh pattern. It can also be noticed that with reduction in the radius of the mesh-pattern, without altering the *Re*, the axial distance required for the microparticle to reach the steady state location increases. When the radius of the perforated surface is reduced, the width of the microchannel reduces thereby leading to increased velocity of fluid and axial displacement.

**Figure 8:**
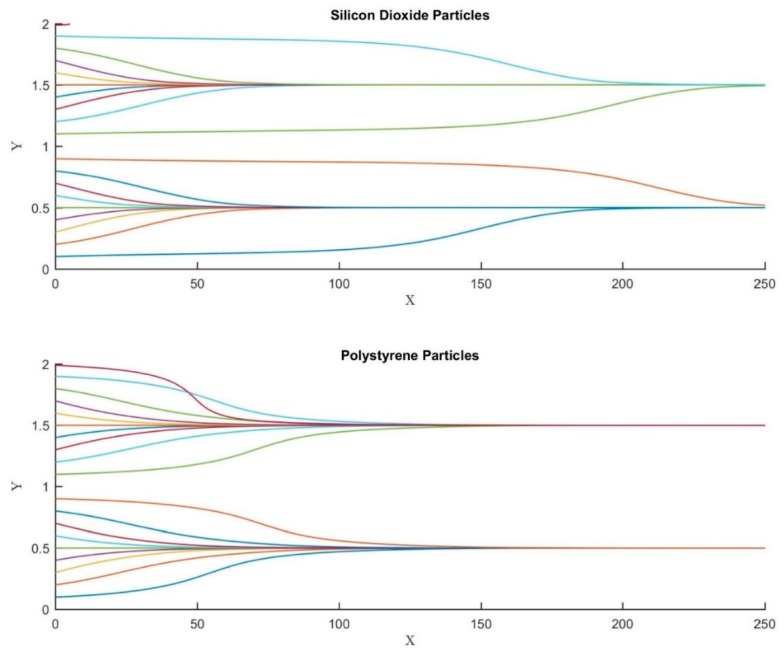
The impact of particle properties on its trajectory. Silicon dioxide ($\rho_{e}=$ 2000 Kg/m^3^ and $\varepsilon_{e}=$ 3.5 F/m) and polystyrene ($\rho_{e}=$ 1060 Kg/m^3^ and $\varepsilon_{e}=$ 2.55 F/m) microparticles. All other simulation parameters are kept the same for both figures. X, Y, and Z are the dimensionless lengths x/L, y/L, and z/Hch, respectively.

Figure 8 demonstrates the efficacy of the microfluidic device in focusing microparticles of different types, i.e., silicon dioxide and polystyrene; all properties of the silicon dioxide are available in Alazzam et al. [41]. Both silicon dioxide and polystyrene microparticles are focused at the same lateral locations when all other conditions are maintained the same as demonstrated in the figure. This demonstrates the efficacy of the microfluidic device in achieving 3D focusing for all types of microparticles. This is extremely important as focusing which is carried out as a prerequisite for separation/focusing is carried out on heterogeneous samples rather than homogeneous samples.

## 4. Conclusions

We have presented a novel technique that aims at sorting/focusing microparticles in fluidic microchannel using continuous and partially perforated electrodes. We have shown that simultaneous 3D focusing at multiple locations along the width of the microchannel is actually possible with a perforated bottom electrode. A comprehensive numerical study with all major parameters involved in the model is reported. It is worth noticing that this model can be used for studying a threshold value for the Reynolds number below which 3D focusing becomes very difficult.

At the transient phase, the initial conditions or positions of the microparticles, the volume, the density and the dielectric property, the medium density, the viscosity and the dielectric property, the channel size and the volumetric flowrate, and the electric field between the electrodes determine the 3D focusing of microparticles at different defined paths within the microchannel.

## Supplementary Materials

The following are available online at https://www.mdpi.com/2079-6374/9/3/99/s1, Model Validation. The mathematical model, i.e., Equation (3), is validated against experimental data in this section. For this, a microfluidic device with a single set of electrodes at one side of the microchannel is used. A number of electrodes are deployed on one side of the microchannel in order to separate selected cells form a mixture [29,42]. The sample preparation, design details, and the fabrication process are detailed in [29,42]. The trajectory of single Red Blood Cells (RBCs) under the effect of nDEP is recorded using a high-speed camera. The actual path of single blood cells is presented in Figure S1. The model is used to predict the trajectory of a single microparticle with similar properties as RBCs and under similar operating conditions. Figure S1 displays the comparison between the trajectories of single blood cell during experiment, and the one obtained using the numerical model. Very good agreement between the experimental and the predicted trajectory is observed thereby validating our numerical model.

## References

[1] Huh D., Gu W., Kamotani Y., Grotberg J.B., Takayama S. (2005). Microfluidics for flow cytometric analysis of cells and particles. Physiol. Meas..

[2] Simonnet C., Groisman A. (2006). High-throughput and high-resolution flow cytometry in molded microfluidic devices. Anal. Chem..

[3] Wheeler A.R., Throndset W.R., Whelan R.J., Leach A.M., Zare R.N., Liao Y.H., Farrell K., Manger I.D., Daridon A. (2003). Microfluidic device for single-cell analysis. Anal. Chem..

[4] Oh S.-H., Wood D.K., Lee S.-H., Dane K.Y., Daugherty P.S., Soh H., Cleland A. Micromachined Broadband RF Cytometer for High-Throughput Analysis of Mammalian Cells. Proceedings of the MicroTAS.

[5] Holmes D., Morgan H., Green N.G. (2006). High throughput particle analysis: Combining dielectrophoretic particle focussing with confocal optical detection. Biosens. Bioelectron..

[6] Wood D., Requa M., Cleland A. (2007). Microfabricated high-throughput electronic particle detector. Rev. Sci. Instrum..

[7] Rodriguez-Trujillo R., Castillo-Fernandez O., Garrido M., Arundell M., Valencia A., Gomila G. (2008). High-speed particle detection in a micro-Coulter counter with two-dimensional adjustable aperture. Biosens. Bioelectron..

[8] Cheung K.C., Di Berardino M., Schade-Kampmann G., Hebeisen M., Pierzchalski A., Bocsi J., Mittag A., Tárnok A. (2010). Microfluidic impedance-based flow cytometry. Cytom. Part A.

[9] Arora A., Simone G., Salieb-Beugelaar G.B., Kim J.T., Manz A. (2010). Latest developments in micro total analysis systems. Anal. Chem..

[10] Weibel D.B., Whitesides G.M. (2006). Applications of microfluidics in chemical biology. Curr. Opin. Chem. Biol..

[11] Kale A., Patel S., Xuan X. (2018). Three-dimensional reservoir-based dielectrophoresis (rDEP) for enhanced particle enrichment. Micromachines.

[12] Gauthier V., Bolopion A., Gauthier M. (2017). Analytical formulation of the electric field induced by electrode arrays: Towards automated dielectrophoretic cell sorting. Micromachines.

[13] Mathew B., Alazzam A., Khashan S., Abutayeh M. (2017). Lab-on-chip for liquid biopsy (LoC-LB) based on dielectrophoresis. Talanta.

[14] Mathew B., Alazzam A., Khashan S., El-Khasawneh B. (2016). Path of microparticles in a microfluidic device employing dielectrophoresis for hyperlayer field-flow fractionation. Microsyst. Technol..

[15] Kim M., Jung T., Kim Y., Lee C., Woo K., Seol J.H., Yang S. (2015). A microfluidic device for label-free detection of Escherichia coli in drinking water using positive dielectrophoretic focusing, capturing, and impedance measurement. Biosens. Bioelectron..

[16] Chiriacò M., Bianco M., Nigro A., Primiceri E., Ferrara F., Romano A., Quattrini A., Furlan R., Arima V., Maruccio G. (2018). Lab-on-chip for exosomes and microvesicles detection and characterization. Sensors.

[17] Stiharu I., Alazzam A., Nerguizian V., Roman D. (2015). Single living cell manipulation and identification using microsystems technologies. Microsyst. Nanoeng..

[18] Liu X., Barizuddin S., Shin W., Mathai C.J., Gangopadhyay S., Gillis K.D. (2011). Microwell device for targeting single cells to electrochemical microelectrodes for high-throughput amperometric detection of quantal exocytosis. Anal. Chem..

[19] Primiceri E., Chiriacò M., Notarangelo F., Crocamo A., Ardissino D., Cereda M., Bramanti A., Bianchessi M., Giannelli G., Maruccio G. (2018). Key enabling technologies for point-of-Care diagnostics. Sensors.

[20] Jiang T., Ren Y., Liu W., Tang D., Tao Y., Xue R., Jiang H. (2018). Dielectrophoretic separation with a floating-electrode array embedded in microfabricated fluidic networks. Phys. Fluids.

[21] Xuan X., Zhu J., Church C. (2010). Particle focusing in microfluidic devices. Microfluid. NanoFluid..

[22] Mao X., Lin S.C.S., Dong C., Huang T.J. (2009). Single-layer planar on-chip flow cytometer using microfluidic drifting based three-dimensional (3D) hydrodynamic focusing. Lab Chip.

[23] Destgeer G., Sung H.J. (2015). Recent advances in microfluidic actuation and micro-object manipulation via surface acoustic waves. Lab Chip.

[24] Ahmed H., Destgeer G., Park J., Jung J.H., Sung H.J. (2018). Vertical hydrodynamic focusing and continuous acoustofluidic separation of particles via upward migration. Adv. Sci..

[25] Zhu J., Xuan X. (2009). Dielectrophoretic focusing of particles in a microchannel constriction using DC-biased AC flectric fields. Electrophoresis.

[26] Mathew B., Alazzam A., El-Khasawneh B., Maalouf M., Destgeer G., Sung H.J. (2015). Model for tracing the path of microparticles in continuous flow microfluidic devices for 2D focusing via standing acoustic waves. Sep. Purif. Technol..

[27] Cheng I.-F., Chang H.-C., Hou D., Chang H.-C. (2007). An integrated dielectrophoretic chip for continuous bioparticle filtering, focusing, sorting, trapping, and detecting. Biomicrofluidics.

[28] Cheng I.-F., Froude V.E., Zhu Y., Chang H.-C., Chang H.-C. (2009). A continuous high-throughput bioparticle sorter based on 3D traveling-wave dielectrophoresis. Lab Chip.

[29] Alazzam A., Mathew B., Khashan S. (2017). Microfluidic platforms for bio-applications. Advanced Mechatronics and MEMS Devices II.

[30] Nerguizian V., Stiharu I., Al-Azzam N., Yassine-Diab B., Alazzam A. (2019). The effect of dielectrophoresis on living cells: Crossover frequencies and deregulation in gene expression. Analyst.

[31] Pethig R. (2017). Where is dielectrophoresis (DEP) going?. J. Electrochem. Soc..

[32] Yin D., Zhang X., Han X., Yang J., Hu N. (2019). Multi-Stage Particle Separation based on Microstructure Filtration and Dielectrophoresis. Micromachines.

[33] Alhammadi F., Waheed W., El-Khasawneh B., Alazzam A. (2018). Continuous-Flow Cell Dipping and Medium Exchange in a Microdevice using Dielectrophoresis. Micromachines.

[34] Alazzam A., Stiharu I., Bhat R., Meguerditchian A.N. (2011). Interdigitated comb-like electrodes for continuous separation of malignant cells from blood using dielectrophoresis. Electrophoresis.

[35] Wang L., Flanagan L., Lee A.P. (2007). Side-wall vertical electrodes for lateral field microfluidic applications. J. Microelectromech. Syst..

[36] Yu C., Vykoukal J., Vykoukal D.M., Schwartz J.A., Shi L., Gascoyne P.R. (2005). A three-dimensional dielectrophoretic particle focusing channel for microcytometry applications. J. Microelectromech. Syst..

[37] Lin C.-H., Lee G.-B., Fu L.-M., Hwey B.-H. (2004). Vertical focusing device utilizing dielectrophoretic force and its application on microflow cytometer. J. Microelectromech. Syst..

[38] Maxey M.R., Riley J.J. (1983). Equation of motion for a small rigid sphere in a nonuniform flow. Phys. Fluids.

[39] Schwarzkopf J.D., Sommerfeld M., Crowe C.T., Tsuji Y. (2011). Multiphase Flows with Droplets and Particles.

[40] Mathew B., Alazzam A., Abutayeh M., Gawanmeh A., Khashan S. (2015). Modeling the trajectory of microparticles subjected to dielectrophoresis in a microfluidic device for field flow fractionation. Chem. Eng. Sci..

[41] Alazzam A., Hilal-Alnaqbi A., Alnaimat F., Ramesh S., Al-Shibli M., Mathew B. (2018). Dielectrophoresis-based microfluidic devices for field-flow fractionation. Med. Devices Sens..

[42] Alazzam A., Mathew B., Alhammadi F. (2017). Novel microfluidic device for the continuous separation of cancer cells using dielectrophoresis. J. Sep. Sci..

