# Supplementary material for: Dielectrophoresis Multipath Focusing of Microparticles through Perforated Electrodes in Microfluidic Channels
Source: Biosensors (Basel). 2019 Aug 7;9(3):99. doi: 10.3390/bios9030099 (PMC6784380; doi:10.3390/bios9030099)
Supplement: Supplementary file 1 [file biosensors-09-00099-s001.pdf]

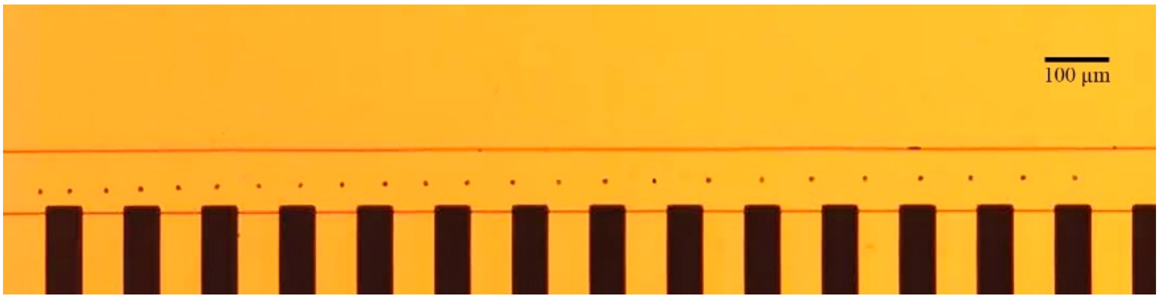

(**a**)

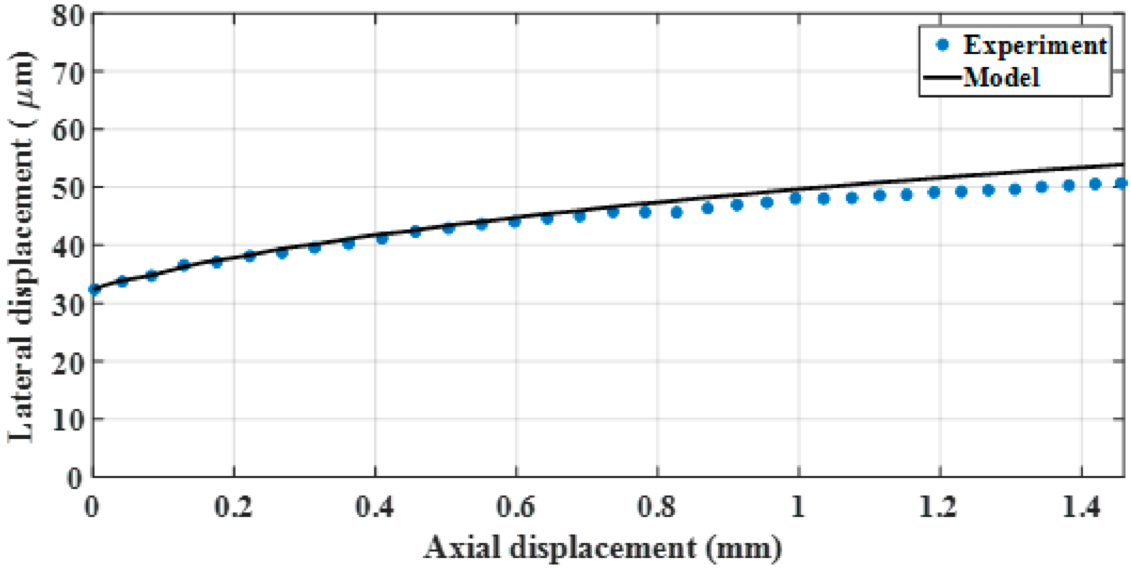

(**b**)

Figure S1. (**a**) Experimental trajectory of single RBC using interdigitated electrodes. The dark rectangles represent the electrodes; (**b**) comparison between the experimental actual path of RBC and the model result (Experiment: electrode width: ~60 µm. electrode gap: ~60 µm, channel width: ~80 µm. channel height: ~30 µm, flow rate: 5 µL/hr, applied voltage and frequency: 10 V_{PP} @10KHz; Model: same operational and geometrical conditions as experiment, particle diameter: 5.6 µm).
